# Effect of density, phonon scattering and nanoporosity on the thermal conductivity of anisotropic cellulose nanocrystal foams

**DOI:** 10.1038/s41598-021-98048-y

**Published:** 2021-09-21

**Authors:** Varvara Apostolopoulou-Kalkavoura, Pierre Munier, Lukasz Dlugozima, Veit-Lorenz Heuthe, Lennart Bergström

**Affiliations:** Department of Materials and Environmental Chemistry, Stockholm University, 10691 Stockholm, Sweden

**Keywords:** Nanoparticles, Surfaces, interfaces and thin films, Characterization and analytical techniques, Materials science

## Abstract

Anisotropic cellulose nanocrystal (CNC) foams with densities between 25 and 130 kg m^−3^ (CNC_25_ –CNC_130_) were prepared by directional ice-templating of aqueous dispersions. Estimates of the solid and gas conduction contributions to the thermal conductivity of the foams using a parallel resistor model showed that the relatively small increase of the radial thermal conductivity with increasing foam density can be attributed to interfacial phonon scattering. The foam wall nanoporosity and, to a lesser extent, the orientation of the CNC particles and alignment of the columnar macropores, also influence the insulation performance of the foams. The insight on the importance of phonon scattering for the thermal insulation properties of nanocellulose foams provides useful guidelines for tailoring nanofibrillar foams for super-insulating applications.

## Introduction

Biobased materials for thermal insulation and thermal management have received substantial research interest thanks to their low thermal conductivity^1–3^ and potential to reduce the carbon footprint by replacing fossil-derived insulating materials such as polyurethane and polystyrene foams^4,5^. Recent work has also suggested that an increase in the share of biobased materials in buildings could serve as an important carbon sink^6^.

Cellulose nanomaterials (CNMs) display a combination of high strength and flexibility^7^ with an anisotropic thermal conductivity^8^, which can be beneficial for, e.g., insulation and thermal management of small devices^9^. While traditional cellulose-based insulation materials, e.g. cavity fillers, are moderately efficient, with thermal conductivities around 40–50 mW m^−1^ K^−1^^10^, CNM-based foams and aerogels can display thermal conductivities substantially below the value for air^11–15^. Recent work on ice-templated anisotropic cellulose nanofibril (CNF)-based foams has shown that the thermal conductivity perpendicular to aligned CNFs depends on both the diameter of the particles and the moisture-controlled swelling of the foam walls^16^. Moisture-induced effects are complex and involve both a reduction of the thermal boundary conductance by the increase in the separation distance between the oriented nanofibrils with uptake of water, and an increase in the thermal conductivity when air is replaced with water in the foam walls^16^. Studies on CNM-based isotropic foams and aerogels revealed a complex and non-linear relationship between density and thermal conductivity^12,14,17,18^, which suggests that other parameters related to the structure and morphology of the foams and aerogels, and the nanostructure of the nanofibrillar pore/cell walls, probably have a strong impact on the thermal conductivity. The lack of a detailed structural characterization and relatively narrow explored density ranges in previous studies have impeded a better understanding of the thermal conductivity of CNM foams and aerogels.

Here, we have investigated the density dependence of the thermal conductivity of anisotropic CNC foams, and discussed the heat transfer contributions by including structural and morphological features of the foams in volume-weighted models. The relative importance of foam density, CNC particle orientation, foam wall nanoporosity and macropore alignment are discussed, and phonon scattering at the interfaces between aligned CNCs is identified as the main reason for the low thermal conductivity of the CNC foams perpendicular to the freezing direction.

## Results and discussion

### Preparation, structure and porosity of freeze-cast CNC foams

**Figure 1:**
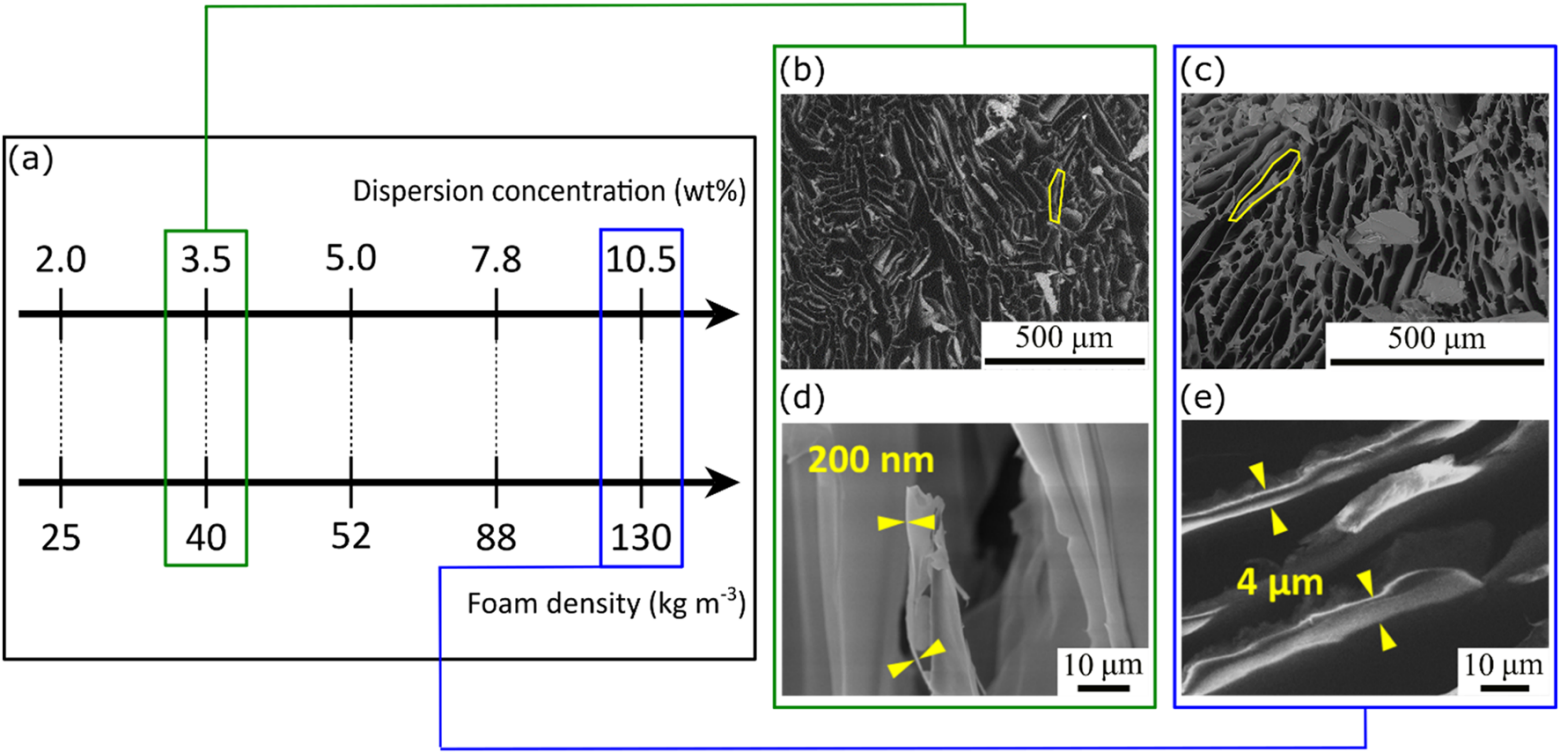
Anisotropic CNC foams prepared by freeze-casting of aqueous dispersions; (**a**) CNC dispersion concentration and corresponding foam density ranges studied herein. Scanning Electron Microscopy (SEM) images of cross-sections of the columnar macroporous structures for foams with densities of; (**b**) 40 kg m^−3^; and; (**c**) 130 kg m^−3^. High-resolution Scanning Electron Microscopy (HRSEM) images of foam walls observed in freeze-cast and freeze-dried foams with densities of; (**d**) 40 kg m^−3^; and; (**e**) 130 kg m^−3^. The foam wall thicknesses are indicated by yellow arrows.

We have prepared anisotropic foams from cellulose nanocrystal (CNC) dispersions by ice templating, or freeze-casting, followed by freeze-drying. CNCs offer a wider dispersion concentration range than for instance CNF dispersions, which gel at relatively low solid contents^19^ and become highly viscous and difficult to process. The density of freeze-cast and freeze-dried foams is directly related to the solid content of the initial liquid dispersions and we have covered a dispersion concentration range from 2.0 to 10.5 wt% (Fig. 1a). More dilute dispersions do not lead to self-standing foams without the addition of binders or additives, while more concentrated dispersions become difficult to process due to their high viscosity. The correlation between the dispersion concentrations and the foam densities is provided in Supplementary Table S1, and the foams will be referred to, as specified in the right-most column, with an acronym specifying their dry density (in kg m^−3^).

The foams were produced using CelluForce CNCs, with diameters of 4.3 ± 0.8 nm and lengths of 173 ± 41 nm (Supplementary Fig. S1), which corresponds to an average aspect ratio around 40. The CNCs possess sulfate half-esters as surface groups and a surface charge of 0.31 ± 0.01 mmol OSO_3_^–^ g^–1^.

The macropores, i.e. the pores that are enclosed by the ice-templated foam walls, display a columnar form due to the unidirectional ice growth (Supplementary Fig. S2), with elongated cross-sections (Fig. 1b, c). The porosity of the CNC foams, which were gravimetrically determined in a moisture-free atmosphere, ranged from 98.3 down to 91.3%, corresponding to foams with densities of 25 and 130 kg m^−3^, respectively. The foam wall thicknesses increased with increasing density, ranging from a few hundred nanometers for the low density (25–40 kg m^−3^) foams (Fig. 1d) to several micrometers for the high density (130 kg m^−3^) foams (Fig. 1e).

**Figure 2:**
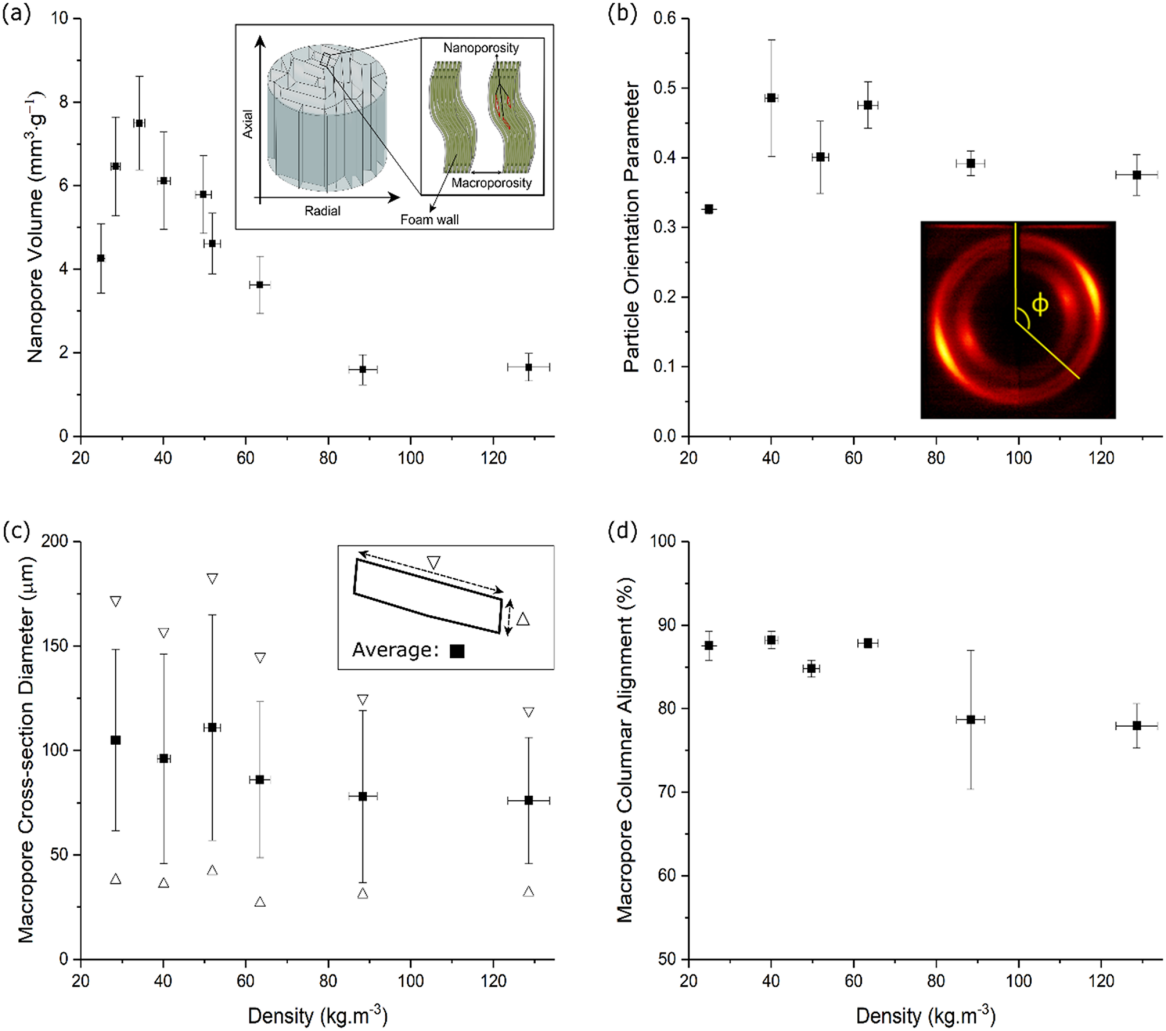
Porosity and alignment in CNC foams. (**a**) Nanopore volume determined by N_2_ adsorption isotherms in CNC foam walls as a function of foam density. *Inset* Schematic illustration of the structure of the anisotropic foam and the foam walls. (**b**) Particle orientation parameter in CNC foams as a function of foam density. *Inset* Typical X-ray diffraction (XRD) 2D-pattern of a CNC foam (the angle $\phi$ depicted in the image is defined in the “Methods” section). (**c**) Macropore cross-section diameter as a function of foam density (the hollow triangles refer to the lengths and widths of the elongated macropore cross-sections, the filled squares refer to the average of both dimensions). (**d**) Macropore columnar alignment estimated by SEM image analysis as a function of foam density.

Freeze-casting orients anisotropic particles in the freezing direction^20^, which from here on will be referred to as the axial direction, with the radial direction referring to the direction perpendicular to the freezing direction (Fig. 2a, inset). The foams possess a hierarchical porous structure having not only macropores but also nanopores within the foam walls, as shown by nitrogen sorption measurements (Fig. 2a).

The nanopore volume of the CNC foams increases with increasing density up to a foam density of 34 kg m^−3^ (CNC_34_ foams), followed by a decrease in the nanopore volume with increasing density for denser foams; from 7.5 mm^3^ g^−1^ for CNC_34_ foams to about 1.6 mm^3^ g^−1^ for CNC_88_ foams and above. The maximum nanopore volume correlates to the concentration range for the onset of formation of a cholesteric phase in a CNC aqueous dispersion^21^. The decrease in the nanopore volume with increasing foam density can be related to a denser packing of the CNC particles as freeze-casting is performed at dispersion concentrations with increasing amounts of cholesteric phase^22,23^. It is interesting to note that even the CNC foams with the highest nanoporosity, CNC_34_, display a four times lower nanopore volume compared to freeze-cast CNF foams^24^ where dense packing is hindered by the entanglement of the kinked and flexible nanofibrils.

The orientation of the (partially) crystalline CNC particles in the foams can be estimated by XRD measurements via the extraction of the particle orientation parameter, also called Hermans’ orientation parameter ($\overline{P}_{2}$). The particle orientation parameter in the CNC foams fluctuates and ranged between $\overline{P}_{2} = 0.49$ for foams with an intermediate density (CNC_40_ foams), to $\overline{P}_{2} \ge 0.37$ for foams with higher densities, and $\overline{P}_{2} = 0.32$ for the CNC_25_ foams (Fig. 2b). The low particle orientation for the foam with the lowest density (CNC_25_) and thinnest foam walls may be related to a less effective collective alignment process.

The macropore cross-section diameter (Fig. 2c) was essentially unaffected by the foam density, and the macroporous columnar alignment (Fig. 2d) decreased only slightly with increasing foam density, which suggests that the CNC particles have a minor effect on the growth of the ice crystals during freeze-casting.

### Thermal conductivity of CNC foams and volume-weighted estimates

The anisotropic thermal conductivity was measured in a customised Hot Disk setup where the temperature and RH could be controlled^25^.

The relative uncertainty of the radial thermal conductivity (λ_r_) values was estimated to be 12% by a propagation analysis of the uncertainties of the parameters required for their calculation, namely the radial thermal diffusivity (α_r_) (Supplementary Fig. S3), the density (ρ) (Supplementary Table S1) and the specific heat capacity (C_p_) (Supplementary Fig. S4), following Eq. (1):

1 $$\uplambda _{r} = \upalpha _{r} \uprho C_{p}$$

The propagation of uncertainly follows Eq. (2):

2 $$\Delta\uplambda _{r} = \uplambda _{r} \sqrt {\left( {\frac{{\Delta \upalpha _{r} }}{{\upalpha _{r} }}} \right)^{2} + \left( {\frac{{\Delta\uprho }}{\uprho }} \right)^{2} + \left( {\frac{{\Delta C_{p} }}{{C_{p} }}} \right)^{2} }$$

where ${\Delta X}$ is the total uncertainty of the X variable, which is a sum of the random and systematic uncertainties^26^. The random uncertainties of α_r_, ρ and C_p_ were based on estimates of the average relative standard deviations (SD) obtained from repeated measurements on several specimens (at least four per specimen for ρ, at least five per specimen for α_r_ and five in total for C_p_), and were multiplied with 1.65 that relates to a 95% confidence interval^26^. The obtained relative random uncertainties were 6%, 4% and 1% for α_r_, ρ and C_p_, respectively. The systematic uncertainty or instrumental bias of ${\upalpha }_{r}$ was estimated to be 5%^27^, while no systematic uncertainty was considered for ${\uprho }$ and $C_{p}$. ${\Delta }C_{p}$ also incorporates the uncertainty on the measurement of water uptake at different RH, which was used to determine C_p_ at various RH using the rule of mixtures.

**Figure 3:**
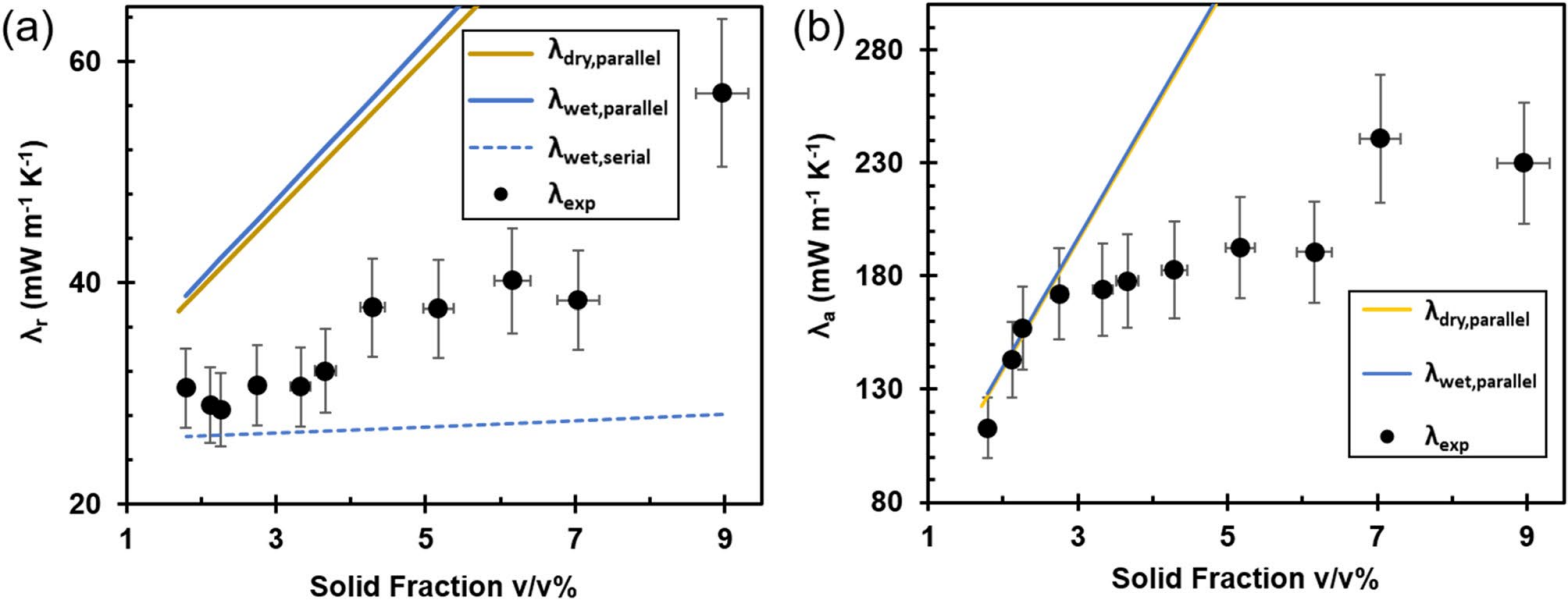
Thermal conductivity of CNC foams as a function of solid fraction (v/v%). (**a**) Radial (λ_r_) and; (**b**) axial (λ_a_) thermal conductivity of CNC foams as a function of CNC solid fraction (v/v%) at 295 K at 50% RH. The black filled circles correspond to experimental data, while the solid lines correspond to volume-weighted parallel sum-based theoretical estimates of the thermal conductivities, at; (i) dry (λ_dry,parallel_) and (ii) 50% RH wet conditions (λ_wet,parallel_), and the dotted line corresponds to the serial addition for 50% RH wet conditions (λ_wet,serial_).

The density dependence of the radial thermal conductivity (λ_r_) and the axial thermal conductivity (λ_a_) of CNC foams, at 50% RH, are shown in Fig. 3. The λ_r_, perpendicularly to the oriented fibrils (Fig. 3a), was between four and six times smaller than the λ_a_ (Fig. 3b). The λ_r_ was relatively unaffected by RH but the λ_a_ increased with increasing RH (Supplementary Fig. S5). The main part of the analysis and discussion will be devoted to the radial thermal conductivity at 50% RH but similar trends are observed at 5, 20 and 80% RH (Supplementary Fig. S6).

Figure 3a shows that, at 50% RH, λ_r_ remained in the range of 28–32 mW m^−1^ K^−1^ for foams with dry solid fractions between 1.7 and 3.5%, which corresponds to dry densities between 25 and 52 kg m^−3^ (see Supplementary Table S1), and increased with increasing solid fraction for foams with dry solid fractions above 3.5%, up to 57 mW m^−1^ K^−1^ for the CNC foams with the highest solid fraction (CNC_130_ foams). The axial thermal conductivity, λ_a_, displayed a pronounced increase with increasing solid fraction for low solid fraction foams (up to CNC_40_ foams), followed by a less steep increase for the high solid fraction foams (Fig. 3b).

The thermal conductivity of the porous foams has been estimated by the so-called parallel resistor model with volume fraction-weighted sums of the solid and gas contributions to the heat transport. The parallel resistor model assumes a simultaneous (parallel) heat transfer through the solid and gas phases^28^. The foam walls of the CNC foams provide connected solid conduction pathways in both the axial and radial directions (see inset in Fig. 2a), which suggests that the parallel resistor model is appropriate to model both the radial and axial thermal conductivities. The radiation and convection contributions are expected to be insignificant as the temperature is relatively low (295 K), and the pore size of the foams is smaller than 1 mm, respectively. The foam walls were considered to consist of densely packed CNC particles that are fully aligned in the axial direction. The thermal conductivity of CNC particles (λ_cell_), perpendicular to (720 mW m^–1^ K^–1^) and along (5700 mW m^–1^ K^–1^) their long axis were used as an approximation of the solid contribution in the radial and axial direction, respectively^8^. The thermal conductivity of air at 295 K (λ_air_ = 25.7 mW m^–1^ K^–1^) was used for the gas contribution. Using the parallel resistor model, the thermal conductivity for foams with dense walls at dry conditions, λ_dry_, can be expressed as:

3 $$\lambda_{dry, parallel} = \phi_{air} \cdot \lambda_{air} + \phi_{cell} \cdot \lambda_{cell}$$

where $\phi_{air}$ and $\phi_{cell}$ are the volume fractions of air and foam walls, respectively, normalized to the total apparent volume of the foam ($\phi_{air}$ + $\phi_{cell}$ = 1). $\phi_{air}$ and $\phi_{cell}$ were estimated from the apparent density of the foam and the skeletal density of cellulose (see “Materials and methods”).

CNC foams take up water and the contribution of the water content at 50% RH (Supplementary Fig. S7) with λ_H2O_ = 600 mW m^–1^ K^–1^ was included in the estimate of the thermal conductivity of moisture-containing foams (λ_wet_) by Eq. (4):

4 $$\lambda_{wet,parallel} = \phi_{air} \cdot \lambda_{air} + \phi_{cell} \cdot \lambda_{cell} + \phi_{H2O} \cdot \lambda_{H2O}$$

where $\phi_{H2O}$ is the water volume fraction, obtained from the gravimetrically determined water uptake (see “Materials and methods”). It is interesting to note that the difference between λ_dry_ and λ_wet_ direction is (1–2 mW m^–1^ K^–1^), which suggests that the replacement of air with water has a minor influence on the thermal conductivity of CNC foams at 50% RH.

The thermal conductivity can also be described by the serial resistor model^29–31^. Combinations of the parallel model (described above) and the serial model have been used to fit the thermal conductivity of various isotropic porous materials^31–33^. The serial model involves addition of the different contributions to the thermal conductivity at 50% RH as given by Eq. (5):

5 $$\lambda_{wet,serial} = \frac{1}{{\frac{{\phi_{air} }}{{\lambda_{air} }} + \frac{{\phi_{cell} }}{{\lambda_{cell} }} + \frac{{\phi_{H2O} }}{{\lambda_{H2O} }}}}$$

The serial model, as it assumes a heat transfer pathway that alternates from the solid to the gas phases, results in much lower values compared to the parallel model (Fig. 3a).

Figure 3b shows that the volume-weighted estimates of the gas and solid contributions using the parallel resistor model, Eqs. (3) and (4), corresponded relatively well to the axial thermal conductivity of CNC foams up to a solid fraction of 3.3%, or a density of 50 kg m^−3^, but overestimates the axial thermal conductivity at high foam solid fractions. It should be noted that a reduction of the assigned value of 5.7 W m^−1^ K^−1^ for the solid phase thermal conductivity of cellulose in the axial direction^8^ would improve the fit between the theoretical estimate and the experimental values also for the high solid fraction foams but the non-linear solid fractions dependence of λ_a_ suggests that there are other factors than a possible reduction of the solid phase thermal conductivity that contribute to limit the increase of λ_a_ with increasing density.

### Knudsen effect and phonon scattering

It is well known that the gas conduction is significantly reduced when the pore size becomes similar to or smaller than the mean free path of air molecules, the so-called Knudsen effect^34^. The effect of nanopores on the gas conduction contribution to thermal conductivity, λ_np_, can be estimated by Eq. (6):

6 $$\uplambda _{{{\text{np}}}} = \frac{{\uplambda _{air} }}{{1 + 2\upbeta \cdot {\text{Kn}}}}$$

where β is a characteristic number equal to 2 for foams and aerogels and Kn is the Knudsen number, which can be estimated by dividing the mean free path of air molecules by the pore size^34^. The nanoporosity of the foam walls at 50% RH varied between 5 and 8% (this range varies at other RH due to different swelling percentages, see Supplementary Fig. S7), and the average nanopore diameters ranged between 7 and 10 nm (Supplementary Table S2).

The high Knudsen number (4–6) in the nanopores results in a λ_np_ that is below 1.5 mW m^–1^ K^–1^ at 0–80% RH for all CNC foams, while thermal conductivity in the much larger macropores, λ_mp_ (calculated with the same formula), is very close to the value for air because the Knudsen effect is negligible at pore sizes above 30 μm (Fig. 2c).

**Figure 4:**
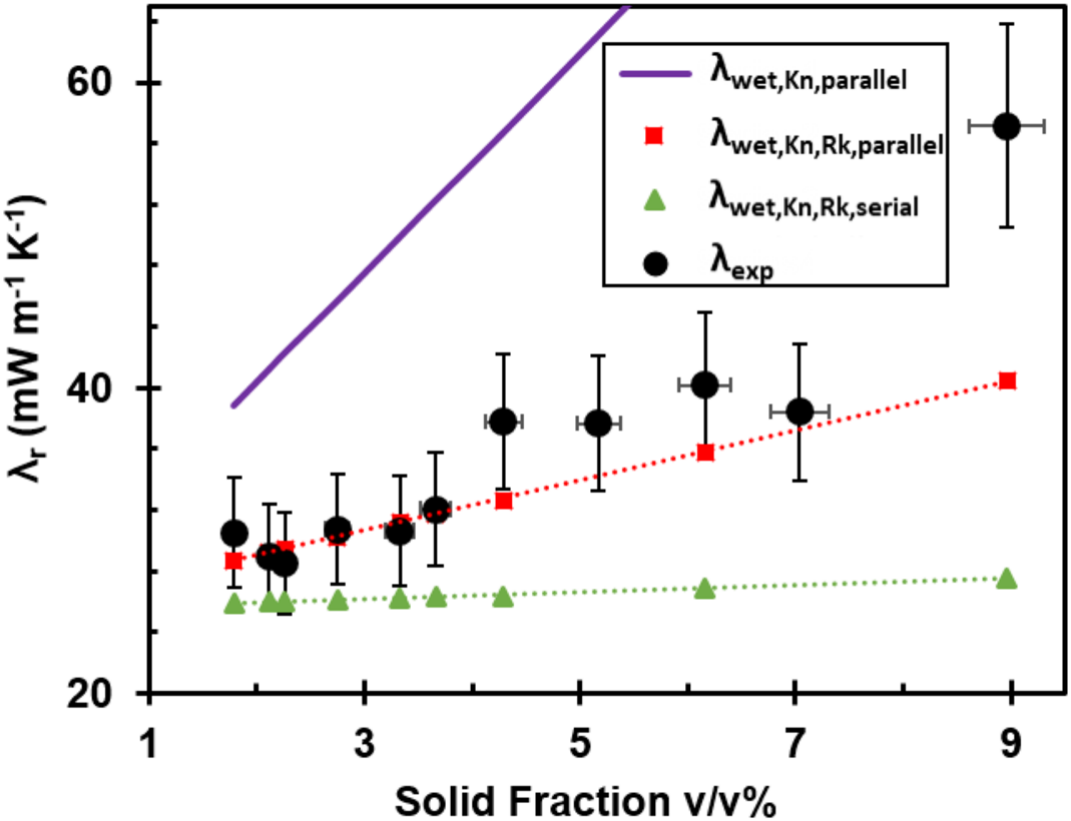
Solid fraction-dependent heat transfer mechanisms in CNC foams. Radial thermal conductivity (λ_r_) of CNC foams as a function of CNC solid fraction (v/v%) including the experimental data at 50% RH, the theoretical estimates of λ_wet,Kn,parallel_ including both the uptake of water at 50% RH and the Knudsen effect in the nanopores, and the theoretical estimates of λ_wet,Kn,Rk,parallel_ and λ_wet,Kn,Rk,serial_ additionally including the particle–particle interfacial effects in the foam walls and distinguishing between parallel and serial addition.

By incorporating the Knudsen effect and introducing separate gas contributions for the macropores (λ_mp_) and the nanopores (λ_np_), we obtain a parallel volume-weighted estimate of the radial thermal conductivity of moisture-containing foams, λ_wet,Kn,parallel_ expressed by Eq. (7):

7 $$\lambda_{wet,Kn, parallel} = \phi_{mp} \cdot \lambda_{mp} + \phi_{np} \cdot \lambda_{np} + \phi_{cell} \cdot \lambda_{cell} + \phi_{H2O} \cdot \lambda_{H2O}$$

where $\phi_{np}$ is estimated from the nanopore volume obtained by N_2_ adsorption measurements (see Fig. 2a) and the total foam volume, while $\phi_{mp}$ corresponds to the remaining volume of air ($\phi_{mp} + \phi_{np} = \phi_{air}$). However, the λ_wet,Kn,parallel_ estimate is much higher than the measured radial thermal conductivity (Fig. 4), which shows that the Knudsen effect is of minor importance due to the small fraction of nanopores in the foams. Nevertheless, λ_r_ attained a value close to the value for air at Π_np_ values above 7% at 50% RH, (Supplementary Fig. S8).

The solid conduction contribution to the thermal conductivity of nanostructured materials can be substantially reduced by phonon scattering at particle–particle interfaces^16^. The effect of phonon scattering at interfaces can be expressed by the interfacial thermal resistance, or Kapitza resistance (R_k_), which can be estimated by Eq. (8):

8 $$R_{k} = \frac{{g_{i} }}{{\lambda_{i} }} = { }\frac{{d_{t} }}{{\lambda_{t} }}{ } - { }2 \cdot \frac{d}{{\lambda_{cell} }}$$

where g_i_ is the surface separation distance between two adjacent CNC particles, λ_i_ is the interfacial thermal conductivity, *d* is the average diameter of a CNC particle (= 4.3 ± 0.8 nm), and λ_t_ and d_t_ are the thermal conductivity and length, respectively, of a system consisting of two CNC particles placed parallel to each other with a gap, g_i_. The interfibrillar separation distance g_i_ has been shown to depend on the water uptake and was estimated to vary between 5.1 and 6.2 Å in the foam walls of freeze-cast CNF foams for RH 35–65%^16^. The water uptake of CNC foams is significantly smaller than for CNF foams^16^ and we have used the calculated value of g_i_ of 2.3 ± 0.4–3.7 ± 0.7 Å at 50% RH (see Supplementary Fig. S7). The value for λ_t_ = 270 mW m^–1^ K^–1^ was obtained from Diaz et al^8^. The interfacial thermal resistance in the radial direction of aligned CNC particles was estimated to 2.2 × 10^–8^ m^2^ K W^−1^. It is interesting to note that the estimated R_k_ for CNC is of a similar magnitude (10^–8^ m^2^ K W^−1^) as for carbon nanotubes^35^.

The effect of phonon scattering at interfaces can be included in an estimate of the (solid) thermal conductivity of a thin film (i.e. the foam wall) of aligned CNC nanoparticles, λ_p_, by Eq. (9):

9 $$\lambda_{p} = \frac{{ \lambda_{cell} }}{{1 + \lambda_{cell} \cdot \frac{{R_{k} }}{d}}}$$

where $\lambda_{cell}$ is the radial thermal conductivity of a single CNC particle (720 mW m^–1^ K^–1^ as mentioned above^8^). Including the estimated interfacial thermal resistance for the CNC foam walls (2.2 × 10^–8^ m^2^ K W^−1^) resulted in an estimated solid contribution to the thermal conductivity in the radial direction, λ_p_, of 158–163 mW m^–1^ K^–1^ at 50% RH. The parallel volume-weighted estimate for the radial thermal conductivity of moisture-containing foams that accounts for both phonon scattering and Knudsen effects, λ_wet,Kn,Rk,parallel_ is given by Eq. (10):

10 $$\lambda_{wet,Kn,Rk,parallel} = \phi_{mp} \cdot \lambda_{mp} + \phi_{np} \cdot \lambda_{np} + \phi_{cell} \cdot \lambda_{p} + \phi_{H2O} \cdot \lambda_{H2O}$$

Figure 4 shows that the obtained λ_wet,Kn,Rk_ estimate corresponds well with the experimental values for the radial thermal conductivities of the CNC_25-88_ foams, which suggests that a significant reduction of the solid conduction by phonon scattering is essential to obtain anisotropic CNC foams with low radial thermal conductivities.

For comparison, we have also estimated the thermal conductivity using the corresponding serial resistor model, given by Eq. (11):

11 $$\lambda_{wet,Kn,Rk,serial} = \frac{1}{{\frac{{\phi_{mp} }}{{\lambda_{mp} }} + \frac{{\phi_{np} }}{{\lambda_{np} }} + \frac{{\phi_{cell} }}{{\lambda_{p} }} + \frac{{\phi_{H2O} }}{{\lambda_{H2O} }}}}$$

which shows that the serial model underestimates the radial thermal conductivity (Fig. 4). Phonon scattering at the solid–gas interfaces^8,36,37^ could also reduce the thermal conductivity, but it was not possible to evaluate the possible magnitude of this phenomenon.

## Conclusions

**Figure 5:**
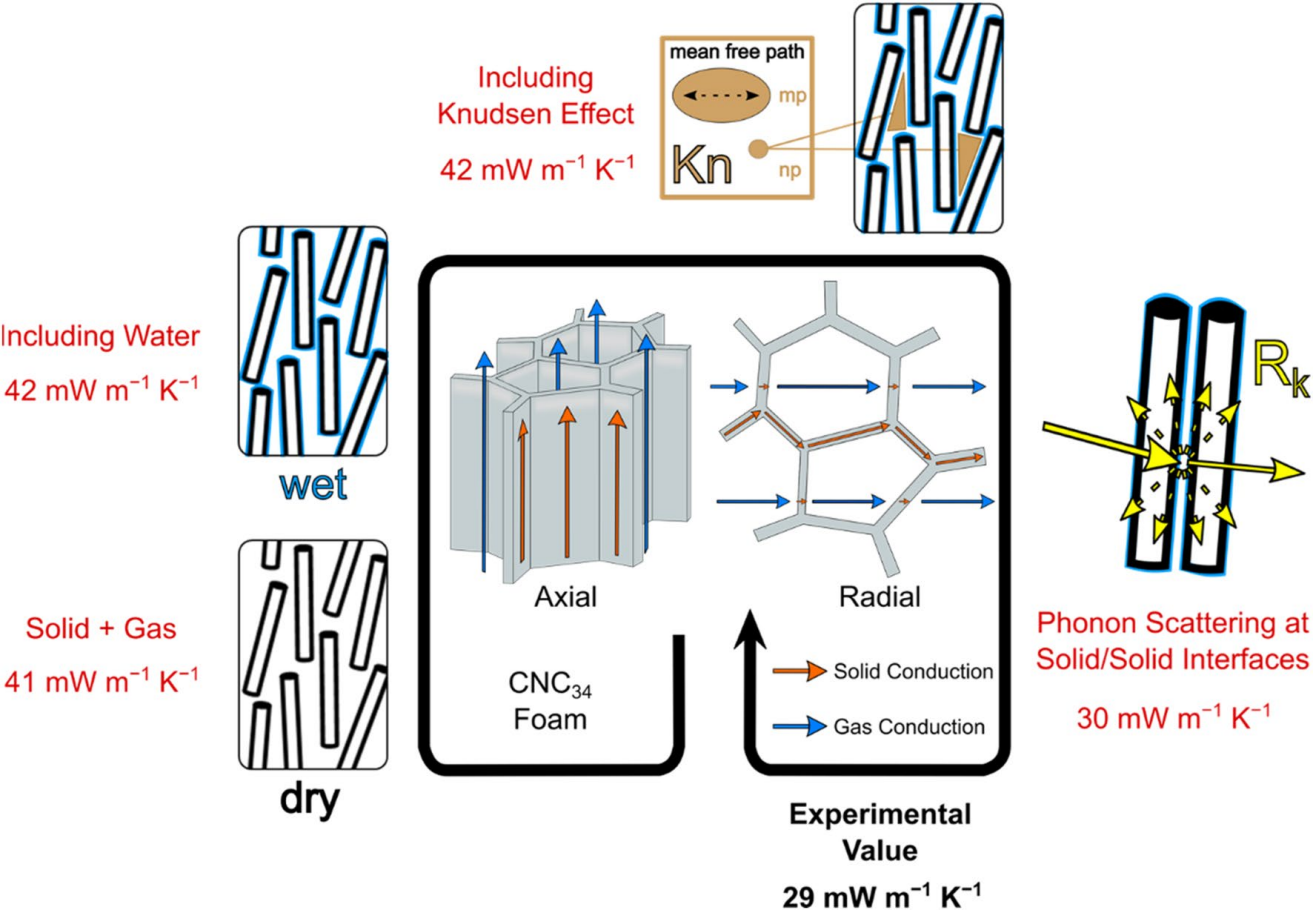
Summary of the assessed heat transfer modes in anisotropic CNC foams. Illustration of the morphological and structural features and the theoretical estimates (red numbers) based on the summation of the solid and gas contributions to the radial thermal conductivity of CNC_34_ foams. The experimentally determined radial thermal conductivity is highlighted in black and bold.

The thermal conductivity of freeze-cast anisotropic CNC foams with densities between 25 and 130 kg m^−3^ were 4–6 times lower perpendicular to (radially) compared to along (axially) the freezing direction. Theoretical estimates based on the main heat transfer contributions to solid conduction, gas conduction and water uptake using a parallel resistor model, showed that the reduction of the solid conduction due to phonon scattering is much more important than the reduction of the gas contribution by the Knudsen effect for reaching radial thermal conductivities as low as 29 mW m^−1^ K^−1^, for the CNC_34_ foams at 50% RH and 295 K (Fig. 5). The theoretical estimates suggest that the presence of water and the nanoporosity have a relatively small influence on the radial thermal conductivity. The orientation of the CNC particles, the alignment of the columnar macropores and the macropore size may also influence the axial and radial thermal conductivities of high density foams.

This study shows that phonon scattering dominates the heat transfer properties of CNC foams, and suggests how nanofibrillar foams with high interfacial thermal resistance and super-insulating properties can be prepared. Additional information regarding phonon scattering at solid–solid and solid-air interfaces are needed for accurate prediction of the insulating performance of biobased, nanofibrillar foams and aerogels.

## Methods

### Preparation of aqueous dispersions

The CNCs used in this study are a commercial product from CelluForce. The CelluForce powder was dispersed as received in DI water using a mechanical stirrer. The initial concentration was 4.5 wt% and the dispersion was later either diluted with DI water or upconcentrated using a rotary evaporator device in order to obtain higher concentrations.

### Preparation of anisotropic foams

Anisotropic CNC foams were prepared by unidirectional ice templating^20,38^ from dispersions having concentrations of 2, 2.5, 3, 3.5, 4, 4.5, 5, 6, 7, 7.8, 8.9 and 10.5 wt%. Teflon molds, with copper bottom plate, 4 cm in diameter and 2.5 cm in height were filled with CNC dispersion and placed in contact with dry ice, giving a cooling rate of 3 K min^–1^. The final dry foams were obtained by ice sublimation at 0.024 mbar and room temperature (RT) for four days using a freeze-dryer (Christ Alpha 1-2LDplus, Germany).

### Particle characterization

AFM (Dimension 3100, Bruker, USA) operated in tapping mode was used to determine the CNC particle dimensions (Supplementary Fig. S1). A droplet of 0.001–0.005 wt% aqueous CNC dispersion was deposited onto freshly cleaved mica substrate and dried at RT.

The charge density (CD) of the CNCs was determined by conductometric titration to be 0.31 ± 0.01 mmol OSO_3_^–^ per gram of cellulose for the CNCs^39^.

### Foams characterization

SEM images of the foams cross-sections were taken using a HITACHI TM-3000 (Germany) using a 5 kV or 15 kV electron beam at magnifications of × 100–250. High-resolution SEM images of the foams wall were taken using a JEOL JSM-7000 (USA) and a 15 kV electron beam at a magnification of × 50–750.

The cross-section diameters of macropores were measured manually using ImageJ on SEM pictures. The local degree of orientation of the columnar macropores was determined by SEM image analysis using the ImageJ plug-in “OrientationJ”^40^. The image analysis calculates the structure tensor of each pixel of coordinates (x; y) in its local neighborhood. The local orientation and isotropy, i.e., coherency and energy, respectively, of every pixel in the image is evaluated by computing the spatial derivatives in x and y directions using a Gaussian gradient. Histograms of the local orientation weighed by the coherency were obtained for each image and they were fitted to a Gaussian function for assessing the orientation index, f. f was calculated using the full width at half-maximum (fwhm) of the Gaussians as shown in Eq. (12):

12 $${\text{f}} = \frac{180 - fwhm}{{180}}$$

The apparent density, *ρ*_*app*_, of the foams was calculated from the mass and the volume (height * πr^2^) of the foams, that had been kept for 3 days at 50% *RH* and 295 K.

The foam porosity ($\phi_{air}$) was determined from the skeletal density of cellulose (*ρ*_*skel*_), chosen as 1500 kg m^−3^^38^, and the apparent foam density (*ρ*_*app*_), according to Eq. (13):

13 $$\phi_{air} = 1 - \frac{{\rho_{app} }}{{\rho_{skel} }}$$

Nitrogen sorption measurements were performed using ASAP 2020 (Micromeritics Instrument Corporation, Nocross, GA, USA). The CNC foams were degassed at 80 °C for 10 h. The adsorption and desorption cumulative volume of nanopores (for diameters between 17 and 3000 Å) and average nanopore diameters were estimated using the Barrett–Joyner–Halenda (BJH) model^41^.

XRD was used to estimate the crystallinity index and the Hermans’ orientation parameter of CNCs in the foams walls.

The crystallinity index was calculated from 1D diffractograms (Supplementary Fig. S9) obtained with a Panalytical X′Pert PRO diffractometer operated with Cu Kα radiation and in Bragg–Brentano diffraction geometry. Pressed foam samples with a mean thickness under 1 mm were mounted on a Si wafer zero-background holder spinning at a constant rate of 30 rpm and diffraction patterns were measured in a 2θ range 5°–50° with 0.0167° step size. The 1D diffractograms were obtained by integrating the 2D patterns using the Segal method^42^ as described in Eq. (14):

14 $$CI\left( \% \right) = \frac{{I_{200} - I_{am} }}{{I_{200} }} \times 100$$

where I_200_ and I_am_ are the intensity values for the (200) peak of crystalline cellulose located at 2θ = 22°–23° and the amorphous background located at 2*θ* = 18°–19°, respectively. Angular values measured with the Mo source were converted to those that would be obtained with a copper (Cu) X-ray source.

For the Hermans’ orientation parameter, a Bruker D8 Venture single-crystal diffractometer equipped with a Photon II detector was used with Cu-Kα radiation, 100 mm detector distance and 180 s acquisition times. The Hermans’ orientation parameter of the CNCs within the foams was estimated from 2D XRD patterns obtained from uncompressed pieces of foam. The Hermans’ orientation parameter, $\overline{P}_{2}$, quantitatively describes the alignment of the CNCs relatively to the freezing direction. $\overline{P}_{2}$ is obtained by azimuthal integration of the (200) peak of cellulose (*θ* = 11.4°) and Eqs. (15) and (16):

15 $$I\left( \phi \right) \cong \mathop \sum \limits_{n = 0}^{2} a_{n} P_{2n} \left( {\cos \phi } \right)$$

16 $$\overline{P}_{2} = \frac{{a_{1} }}{5}$$

Equation (15) is a Legendre series expansion with which the azimuthal integration was fitted. $\phi$ represents a theoretical angle between a nanoparticle’s main direction and the freezing direction of ice crystals during ice templating. This angle can be identified as the azimuthal angle on the 2D XRD pattern. $I\left( \phi \right)$ represents the intensity of the XRD signal at a certain $\phi$ angle. $P_{2n}$ are even Legendre polynomials and $a_{n}$ are the corresponding fitting coefficients. After normalizing the fitting coefficients to $a_{0} = 1$, the Hermans’ orientation parameter is obtained from the $a_{1}$ coefficient according to Eq. (16)^43^.

DSC (Mettler Toledo 820, Sweden) was used to estimate the specific heat capacity at constant pressure (*C*_*p*_) (Supplementary Fig. S4) of CNC foams at RH = 0 under nitrogen atmosphere at temperatures ranging between − 20 and 50 °C with a heating rate of 10 K min^–1^. Aluminum crucibles and lids were used for the DSC measurements. An empty crucible with lid served as the reference, and sapphire was used as a standard. Five independent samples of 8–10 mg of CNC obtained by compressing the foams into the crucibles were performed at the DSC resulting in the average C_*p*_ value of 898 ± 6.2 J kg^–1^ K^–1^. All samples were carefully dried prior to the addition in the crucibles by heating at 105 °C for 24 h and after placing them in the crucibles for 24 additional hours at 105 °C.

The foam wall nanoporosity (*Π*_*np*_) was estimated using Eq. (17):

17 $$\Pi_{np} = 1 - \frac{{\rho_{wall} }}{{\rho_{scel} }}$$

where *ρ*_*wall*_ is the density of the foam wall and *ρ*_*scel*_ is the skeletal density of cellulose (1500 kg/m^3^^38^). The density of the foam wall is estimated by Eq. (18):

18 $$\rho_{wall} = \frac{{m_{wet} }}{{V_{foam} \cdot \left( {1 - \phi_{air} } \right) + V_{np} }}$$

where *m*_*wet*_ is the *RH*-dependent mass of the foam, *V*_*foam*_ is the volume of the foam, $\phi_{air}$ is the total porosity of the foam, and *V*_*np*_ is the nanopore volume in the foam walls. The normalized nanopore volume was calculated by multiplying the nanopore volume measured by nitrogen sorption at *RH* = 0 with the weight of the foam for the specific foams (Fig. 2a).

The *C*_*p*_ of moist foams (Eq. 19) was calculated by the rule of mixtures and the *C*_*p*_ of water, and the ρ was corrected with respect to the $w_{H2O}$ and the volume shrinkage. The volume shrinkage was estimated by measuring the dimensions of the foams after each RH conditioning cycle.

19 $$C_{{P_{wet} }} = \left( {1 - w_{H2O} } \right) \cdot C_{{P_{dry} }} + C_{{P_{{H_{2} O}} }} \cdot w_{H2O}$$

where $w_{H2O}$ is the water content in the foams in mass fraction, *Cp*_*dry*_ is the dry specific heat capacity at constant pressure of the foams measured in the DSC in J kg^–1^ K^–1^and *Cp*_*H2O*_ is the specific heat capacity at constant pressure of water in J kg^–1^ K^–1^.

### Moisture uptake

The water vapor sorption of the CNC foams under controlled RH and T was determined by measuring the weight change using a high-precision balance (BP 210 S, Sartorius, Germany) placed inside a humidity chamber as described previously^25^. Prior to the measurements, the foams were dried at 313 K and 20% RH. The moisture content ($w_{H2O}$) as a function of *RH* (20, 35, 50, 65 and 80%) was assessed at 295 K. Each measurement lasted 6 h to ensure that steady state was reached, and the foam mass was measured every 5 min.

### Thermal conductivity measurement

The thermal conductivities (*λ*, mW m^–1^ K^–1^) of the foams were determined using the TPS 2500 S Hot Disk Thermal Constants Analyzer in the anisotropic mode. The TPS 2500 S Hot Disk Thermal Constants Analyzer has a reported accuracy for isotropic materials of 2–5%^44^ but the accuracy is less for anisotropic, low thermal conductivity materials^27^. The Kapton transient plane sensor (3.2 mm in radius) was placed between two identical foam pieces (diameter: 4.1 ± 0.1 cm; height: 2.4 ± 0.2 cm). Good thermal contact between the sensor and the foams was ensured by putting a small weight (39 g) onto the samples resulting in a contact pressure of 339 N m^2^ ± 0.4^45^. The heating power and the measurement time were 10 mW and 5 s for CNC_25-40_ and 10 mW and 10 s for CNC_50-130_, respectively. The foams were enclosed in a customized cell, allowing the *RH* to be controlled (2–80% *RH*)^25^. Five independent measurements were performed at 15-min intervals for each *RH* at 295 K and each foam pair and every foam was investigated using 2–4 different pair of foams with the same density. The TPS method in the anisotropic mode yields the radial thermal diffusivity (see Supplementary Fig. S3), from which the thermal conductivity is calculated by multiplying with the volumetric heat capacity (*ρ*_*app*_*C*_*p*_). The axial thermal conductivity of the anisotropic foams at different *RH* and *T* were obtained using the software in the Hot Disk as previously described^16^.

The foam shrinkage during thermal conductivity experiments was taken into consideration when assessing the volume change of the foams during the water uptake measurements. The foams were mounted on the customized cell and the volume of the foams was measured after each humidity cycle with a caliper. Taking into consideration the volume shrinkage and the moisture content, the wet density of the foams was calculated and used for the calculations of the thermal conductivity in Hot Disk.

## Supplementary Information

Supplementary Information.

## Data Availability

The datasets generated during and/or analysed during the current study are available in the [Materials Cloud] repository, [10.24435/materialscloud:49-t3].
