# Supplementary material for: Effect of density, phonon scattering and nanoporosity on the thermal conductivity of anisotropic cellulose nanocrystal foams
Source: Sci Rep. 2021 Sep 21;11:18685. doi: 10.1038/s41598-021-98048-y (PMC8455657; doi:10.1038/s41598-021-98048-y)
Supplement: Supplementary file 1 — Supplementary Information. [file 41598_2021_98048_MOESM1_ESM.docx]

Effect of density, phonon scattering and nanoporosity on the thermal conductivity of anisotropic cellulose nanocrystal foams

Varvara Apostolopoulou-Kalkavoura^1,⸸^, Pierre Munier^1,⸸^, Lukasz Dlugozima^1^, Veit-Lorenz Heuthe^1^, and Lennart Bergström^1,*^

^1^Stockholm University, Department of Materials and Environmental Chemistry, Stockholm, 10691, Sweden
^*^lennart.bergstrom@mmk.su.se
^⸸^these authors contributed equally to the work

### Supplementary information

**Supplementary Table S1. Densities of CNC foams associated to the concentrations of the corresponding initial dispersions and the dry solid fraction.**

| Dispersion concentration (wt%) | ρ_dry_ (kg m^-3^) | Reference name | Dry Solid Fraction (v/v%) |
| --- | --- | --- | --- |
| 2 | 24.9 ± 0.4 | CNC_25_ | 1.7 |
| 2.5 | 28.4 ± 1.5 | CNC_28_ | 2.0 |
| 3 | 34.2 ± 1.8 | CNC_34_ | 2.2 |
| 3.5 | 40.1 ± 0.7 | CNC_40_ | 2.6 |
| 4.5 | 49.7 ± 0.6 | CNC_50_ | 3.2 |
| 5 | 51.9 ± 0.2 | CNC_52_ | 3.5 |
| 6 | 63.4 ± 1.8 | CNC_63_ | 4.2 |
| 7 | 70.3 ± 0.0 | CNC_70_ | 4.8 |
| 7.8 | 88.1 ± 6.9 | CNC_88_ | 6.0 |
| 8.9 | 97.2 ± 0.2 | CNC_97_ | 6.5 |
| 10.5 | 130 ± 4.0 | CNC_130_ | 8.7 |


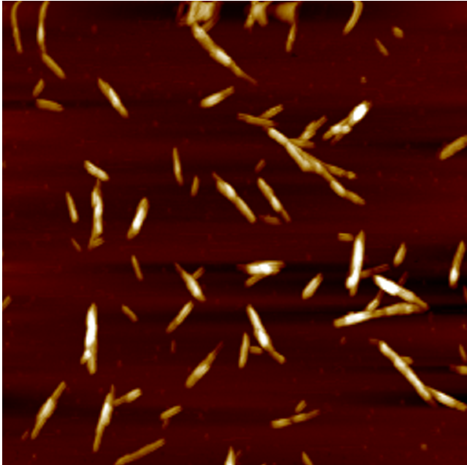


**Supplementary Figure S1.** Determination of CNC particle dimensions. AFM micrograph of a CNC dispersion.

**
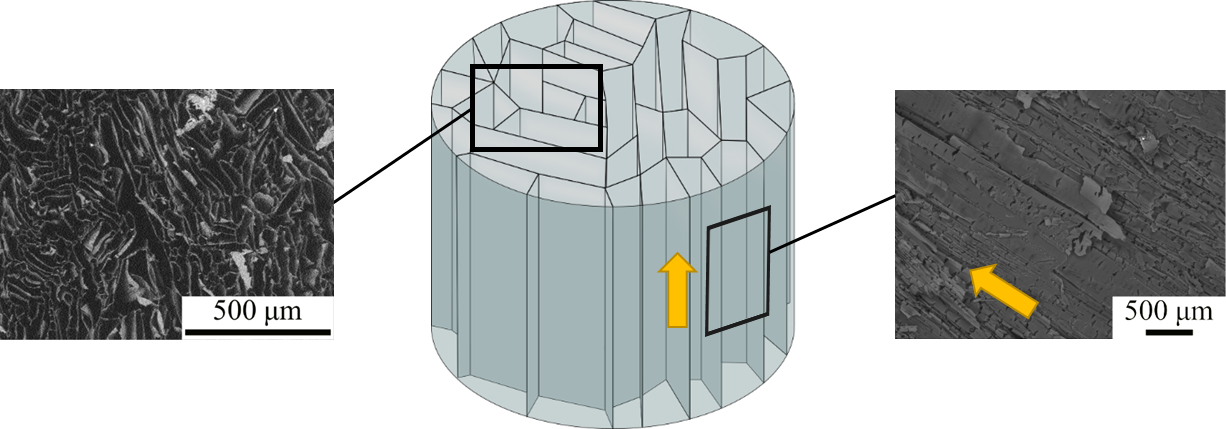
**

**Supplementary Figure S2.** Columnar macroporous structure of freeze-cast foams. Schematic of a freeze-cast and freeze-dried foam with SEM images depicting cross-sectional (left) and longitudinal (right) views.


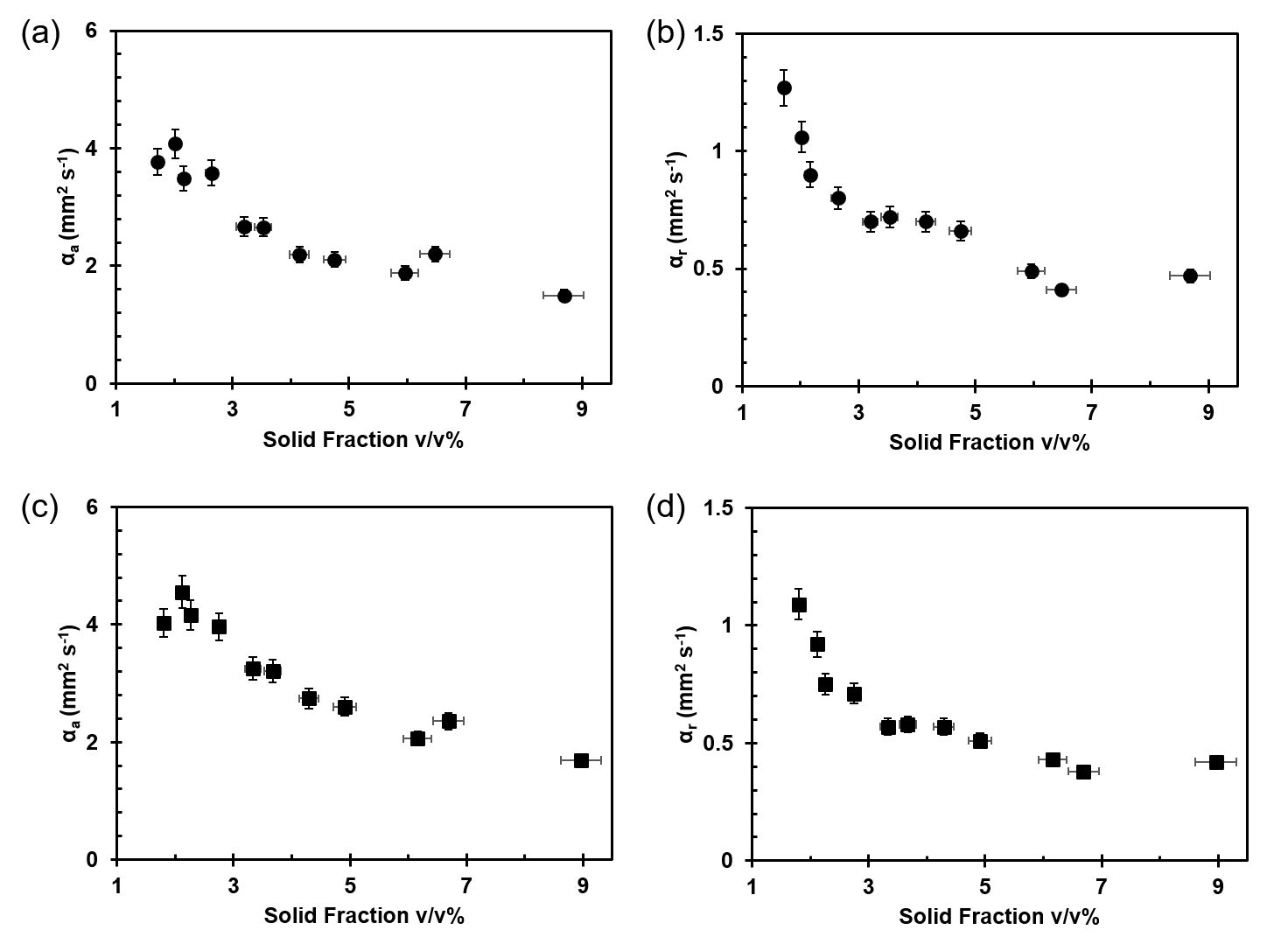


**Supplementary Figure S3.** Thermal diffusivity in the axial (α_a_) and radial directions (α_r_) of the CNC foams, at; (a-b) 5% RH and; (c-d) 50% RH.

**
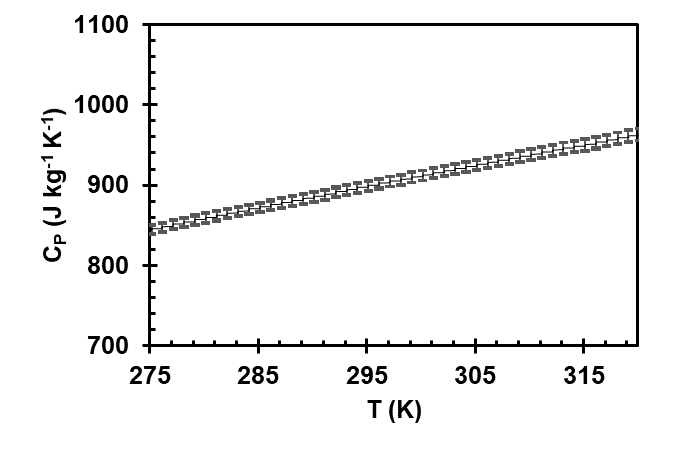
**

**Supplementary Figure S4.** Specific heat capacity at constant pressure (Cp) of CNC foams measured by DSC.


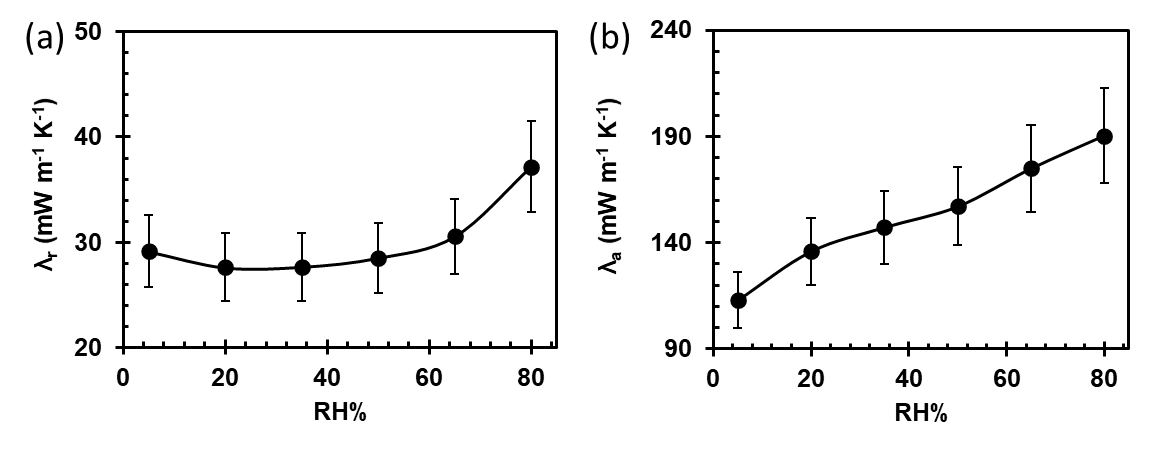


**Supplementary Figure S5.** Thermal conductivity of CNC foams as a function of RH. (a) Radial (λ_r_) and; (b) Axial thermal conductivity (λ_a_) of CNC_34_ foams as a function of RH.

**
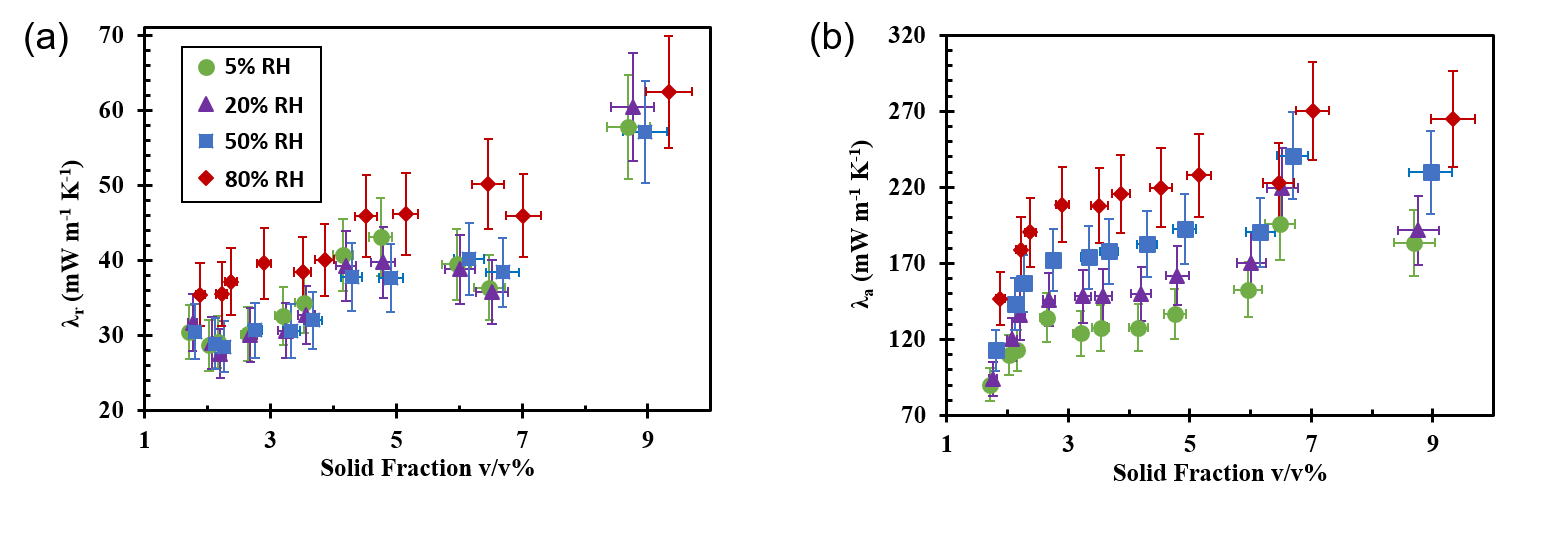
**

**Supplementary Figure S6.** Thermal conductivity of CNC foams at various RH. (a) Radial thermal conductivity (λ_r_) and; (b) Axial thermal conductivity (λ_a_) as a function of solid fraction (v/v%) of CNC foams at 295 K and 5, 20, 50 and 80% RH.

**
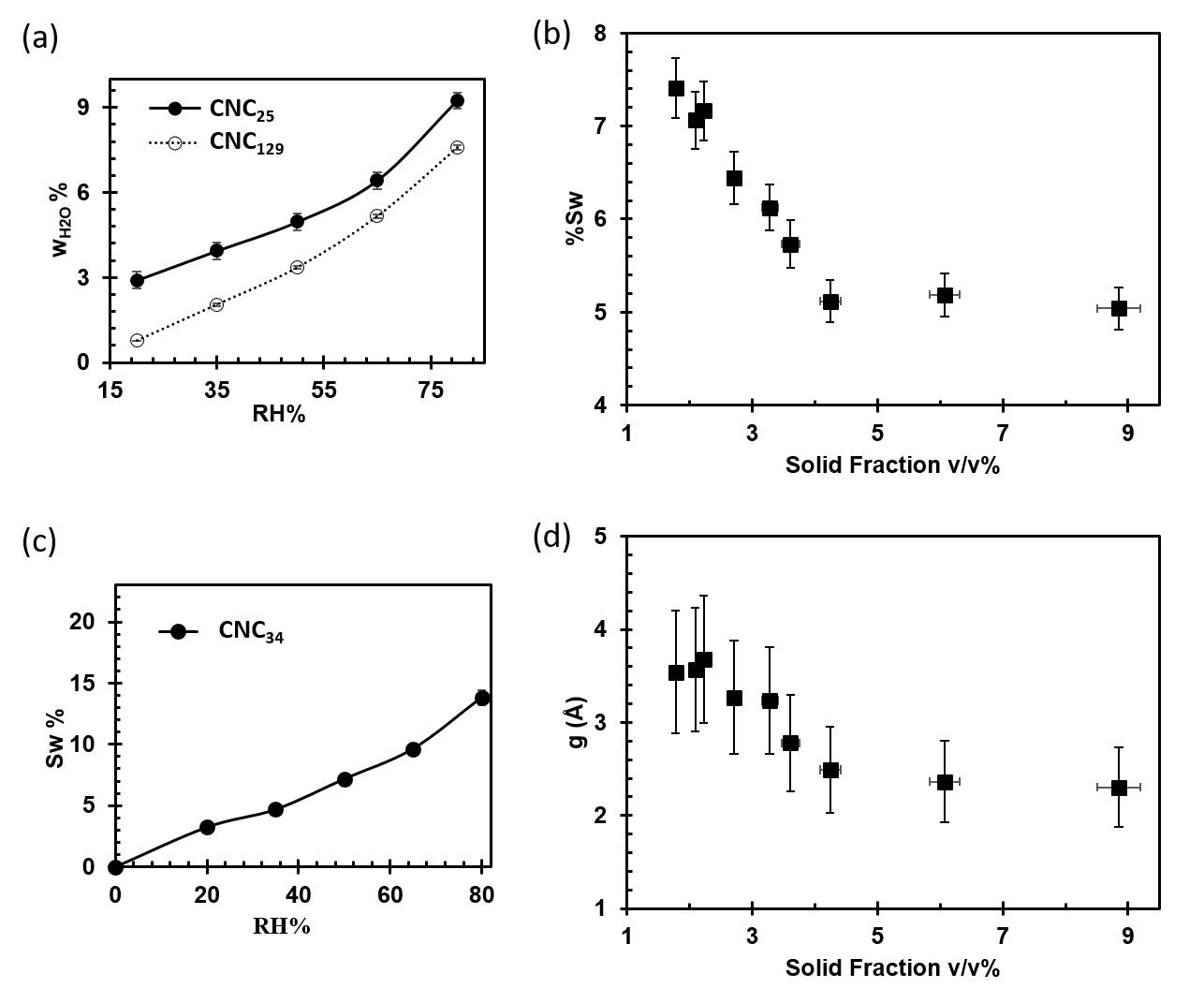
**

**Supplementary Figure S7.** Moisture uptake and swelling in CNC foams. (a) Moisture uptake ($w_{H2O}$) of CNC_25_ and CNC_129_ foams as a function of RH. (b) Swelling as a function of solid fraction (v/v%) for CNC foams. (c) Swelling as a function of RH for CNC_34_ foams. (d) Interparticle distance as a function of CNC solid fraction (v/v%) at 50% RH and 295 K.

**Supplementary Table S2. Barrett-Joyner-Halenda average nanopore diameter in the CNC foams.**

| Foam | Average nanopore diameter (nm) |
| --- | --- |
| CNC_25_ | 9.0 ± 2.1 |
| CNC_28_ | 8.7 ± 2.0 |
| CNC_34_ | 8.2 ± 1.6 |
| CNC_40_ | 9.4 ± 2.1 |
| CNC_50_ | 7.8 ± 1.5 |
| CNC_52_ | 8.5 ± 2.0 |
| CNC_63_ | 8.7 ± 1.9 |
| CNC_88_ | 8.5 ± 2.3 |
| CNC_130_ | 7.2 ± 1.8 |

**
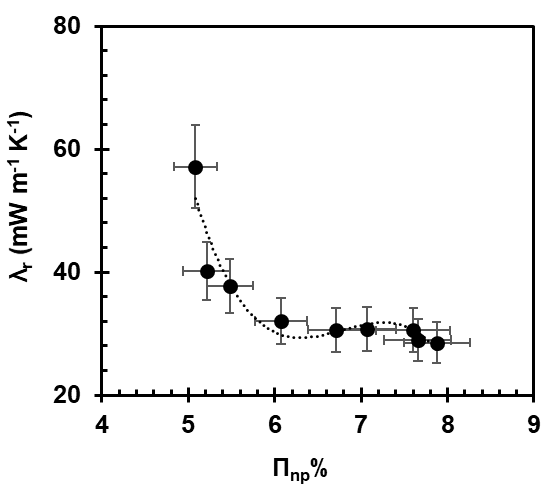
**

**Supplementary Figure S8.** Radial thermal conductivity (λ_r_) of CNC foams as a function of wall nanoporosity (Π_np_%) at 295 K, at 50% RH.


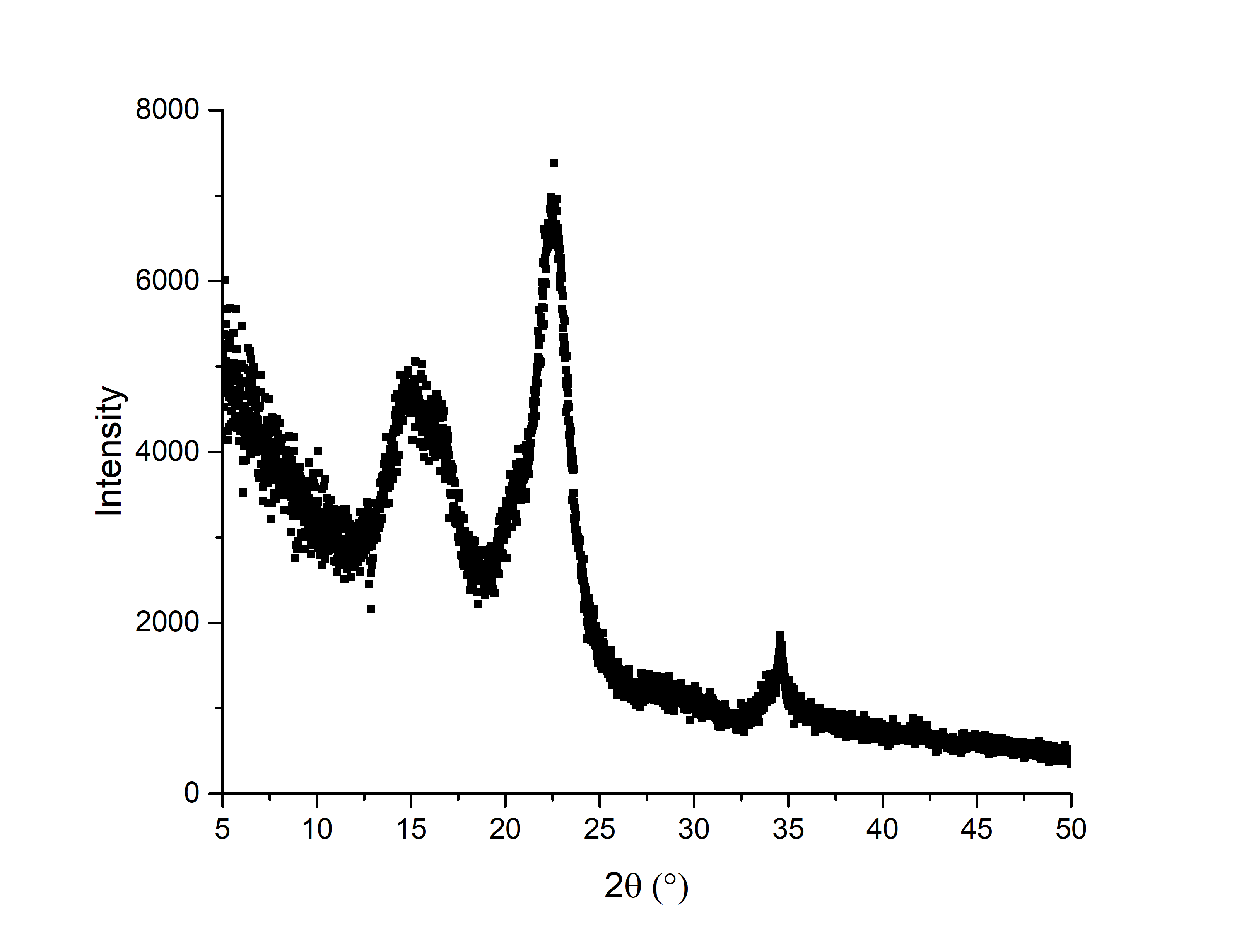


**Supplementary Figure S9.** X-ray diffraction. PXRD 1D spectra obtained for a CNC foam used for determining the crystallinity index.
